# Effects of arginase inhibition on myocardial Ca^2+^ and contractile responses

**DOI:** 10.14814/phy2.15396

**Published:** 2022-07-22

**Authors:** Jin Sun Cho, Young Soo Han, Cole Jensen, Gary Sieck

**Affiliations:** ^1^ Department of Anesthesiology and Pain Medicine Yonsei University College of Medicine Seoul Republic of Korea; ^2^ Department of Physiology and Biomedical Engineering Mayo Clinic Rochester Minnesota USA

**Keywords:** arginase inhibition, Ca^2+^, cardiac contractility, nitric oxide, nitric oxide synthase

## Abstract

Nitric oxide (NO) is thought to increase cardiac contractility by increasing cytosolic Ca^2+^ concentration ([Ca^2+^]_cyt_) during excitation. Alternatively, NO could increase the sensitivity of the contractile response to [Ca^2+^]_cyt_ (Ca^2+^ sensitivity). Arginase regulates NO production by competing with NO synthase (NOS), and thus, arginase inhibition should increase cardiac contractility by increasing NO production. We hypothesized that arginase inhibition increases cardiac contractility by increasing both [Ca^2+^]_cyt_ and Ca^2+^ sensitivity. [Ca^2+^]_cyt_ and contractile (sarcomere length [SL] shortening) responses to electrical stimulation were measured simultaneously in isolated rat cardiomyocytes using an IonOptix system. In the same cardiomyocytes, measurements were obtained at baseline, following 3‐min exposure to an arginase inhibitor (*S*‐[2‐boronoethyl]‐l‐cysteine; BEC) and following 3‐min exposure to BEC plus a NOS inhibitor (*N* ^G^‐nitro‐l‐arginine‐methyl ester; l‐NAME). These responses were compared to time‐matched control cardiomyocytes that were untreated. Compared to baseline, BEC increased the amplitude and the total amount of evoked [Ca^2+^]_cyt_, and the extent and velocity of SL shortening in cardiomyocytes, whereas addition of l‐NAME mitigated these effects. The [Ca^2+^]_cyt_ at 50% contraction and relaxation were not different across treatment groups indicating no effect of BEC on Ca^2+^ sensitivity. The [Ca^2+^]_cyt_ and SL shortening responses in time‐matched controls did not vary with time. Arginase inhibition by BEC significantly increased the amplitude and the total amount of evoked [Ca^2+^]_cyt_, and the extent and velocity of SL shortening in cardiomyocytes, but did not affect Ca^2+^ sensitivity. These effects of BEC were mitigated by l‐NAME. Together, these results indicate an effect of NO on [Ca^2+^]_cyt_ responses that then increase the contractile response of cardiomyocytes.

## Graphical abstract

Arginase inhibition significantly increased the amplitude and total amount of evoked [Ca^2+^]_cyt_ to electrical stimulation. It enhanced myocardial contractility by increasing the extent and velocity of SL shortening in cardiomyocytes, but did not affect Ca^2+^ sensitivity. These effects were mitigated by NOS inhibition, supporting a role of NO on [Ca^2+^]_cyt_ and contractile responses.

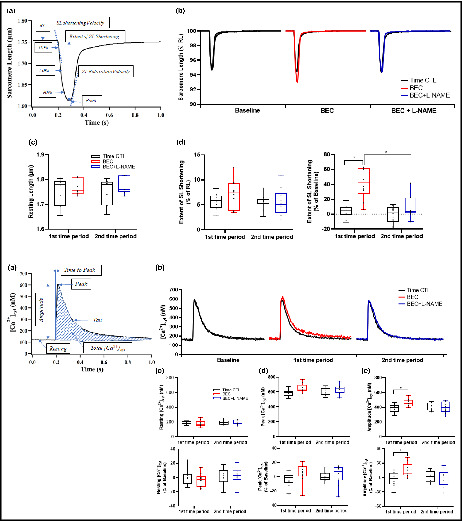

## 1 INTRODUCTION

Nitric oxide (NO) is one of the key molecular players in cardiac contractility via modulation of Ca^2+^ influx critical to excitation–contraction coupling and indirectly by maintaining superoxide balance within the cardiomyocyte (Khan et al., 2003). NO is produced by a family of NO synthases (NOS) (Luo et al., 2014). Under physiological conditions, NOS and arginase compete for l‐arginine to produce NO and l‐ornithine, respectively, whereas when uncoupled from its cofactor or substrate, NOS produces superoxide instead of NO (Luo et al., 2014) This phenomenon is called NOS separation, in which the peroxide produced from unbound NOS reacts with NO to form nitrite peroxide (ONOO^−^). Enhanced generation of both NO and superoxide, and as a consequence, peroxynitrite, has been demonstrated to play critical roles in the etiology of cardiovascular diseases (Khadour et al., 2002; Luo et al., 2014).

Substrate bioavailability is one of the regulatory mechanisms of NOS activity. Arginase competes with NOS for the common substrate l‐arginine to mutually modulate NOS activity, thus regulating NO and reactive oxygen species production. Among NOS isoforms, NOS1 positively modulates cardiac contractility, whereas NOS3 inhibits β‐adrenergic receptor‐mediated increases in cardiac contractility (Hare, 2003). Arginase‐II is the predominant isoform expressed in rodent heart and modulates myocardial contractility by a NOS1‐dependent mechanism (Steppan et al., 2006). Increased arginase expression/activity is associated with reduced NO production and myocardial contractility, which is reversed by arginase inhibition (Khan et al., 2012; Steppan et al., 2006). Previous studies suggested that arginase exerts its effect on myocardial contractility via a NOS‐dependent mechanism by demonstrating that arginase inhibition increases NO bioavailability and restores NO production and NOS coupling (Kim et al., 1985).

The regulatory role of NOS1 on cardiac contractility is constitutively Ca^2+^‐dependent (Heinzel et al., 2008). *NOS1 colocalizes* with the ryanodine receptor (RyR) in the *sarcoplasmic reticulum* (*SR*) (Schulman & Hare, 2012). NOS1 modulates the activation of RyRs, through alterations in the levels of RyR nitrosylation and redox milieu (Steppan et al., 2006). NO‐mediated *S*‐nitrosylation induces RyR activation, increasing cardiac contractility (Khan et al., 2003). *S*‐nitrosylation depends on the redox milieu, and the ratio of superoxide/NO production by NOS is an important determinant of the redox milieu (Sun et al., 2006). NO increases the open possibility of cardiac RyRs (Khan et al., 2003), whereas superoxide irreversibly activates RyR and enhances SR leak, which would increase [Ca^2+^]_cyt_ (Xu et al., 1998). Arginase may influence Ca^2+^ release by regulating NOS1 and its products, superoxide and NO.

The sarco/endoplasmic reticulum Ca^2+^ ATPase (SERCA) and Ca^2+^ sequestration also plays an important role in regulation of [Ca^2+^]_cyt_ (Massion et al., 2003; Primeau et al., 2018). It has been reported that NO stimulates SR Ca^2+^ uptake and thereby can increase SR Ca^2+^ stores, and as a result, increase the total [Ca^2+^]_cyt_ response to electrical stimulation (Ziolo et al., 2008). Thus, both SR Ca^2+^ release and re‐uptake may be affected by arginase inhibition. Although few studies have reported that arginase inhibition increases cardiac contractility (Khan et al., 2012; Steppan et al., 2006), they have not investigated the concurrent changes in [Ca^2+^]_cyt_ and contractility induced by arginase inhibition in cardiomyocytes.We hypothesized that arginase inhibition increases evoked [Ca^2+^]_cyt_ in cardiomyocytes, leading to increased cardiac contractility. Alternatively, arginase inhibition could influence Ca^2+^ sensitivity of cardiomyocytes.

## 2 MATERIALS AND METHODS

### 2.1 Animals

The experimental protocol was approved by the Mayo Clinic Institutional Animal Care and Use Committee (ref #. AA2521‐17‐R19). Studies were performed using 12 (*n* = 6 treatment and *n* = 6 time‐matched controls) male Sprague–Dawley rats weighing 250–350 g (Envigo). The animals were anesthetized with intraperitoneal injection of a mixture of ketamine (90 mg/kg; Zoetis, Inc.) and xylazine (10 mg/kg; Biomed‐MTC; Animal Health, Inc.) and provided 100% O_2_ through a nasal cannula. After confirming the anesthetic status by verifying the lack of a toe pinch reflex withdrawal, a thoracotomy was performed, the heart excised and the animal was euthanized by exsanguination.

### 2.2 Cardiomyocyte isolation

The procedure for isolation of cardiomyocytes from the excised heart has been previously described in detail (Schaible et al., 2016). Briefly, the heart was cannulated via the aorta, connected to a modified Langendorff perfusion system, and perfused with pre‐warmed (37°C) and oxygenated Tyrode's solution for 5 min. The heart was then perfused with a type II collagenase solution for 10 min. The enzymatically digested heart was then minced in a second type II collagenase solution and filtered through a stainless‐steel mesh to isolate cardiomyocytes. The isolated cardiomyocytes were centrifuged at 130 g for 1 min and incubated with Kraft‐Brühe solution at 37°C for 30 min. Then, cardiomyocytes were then incubated in the cardiac myocyte medium at 37°C for another 30 min. With this isolation technique, more than 60% of isolated cardiomyocytes from each heart were viable, as identified by trypan blue exclusion. Cardiomyocytes maintained a rod‐shaped morphology with clear sarcomeric striation patterns and a stable contractile response to field stimulation.

### 2.3 Measurement of simultaneous [Ca^2+^]_cyt_ and contractile responses

**FIGURE 1:**
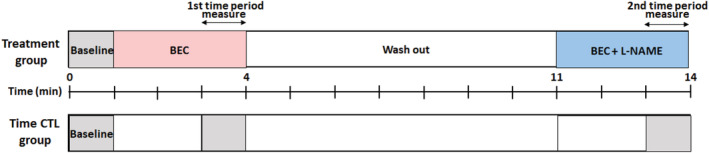
Study design. Cardiomyocytes were perfused with BEC and BEC + l‐NAME for 3 min, respectively, and [Ca^2+^]_cyt_ and sarcomere length (SL) responses of cardiomyocytes were measured during the last minute. The evoked [Ca^2+^]_cyt_ and SL responses were averaged over 10–20 stimulation cycles. Between exposures to BEC and BEC + l‐NAME, the cardiomyocytes were washed by perfusion with Tyrode solution for 7 min. A time‐matched control experiment was performed to determine the effect of time on [Ca^2+^]_cyt_ and SL responses. BEC, *S*‐[2‐boronoethyl]‐l‐cysteine; l‐NAME, *N* ^G^‐nitro‐l‐arginine‐methyl ester.

The [Ca^2+^]_cyt_ and contractile (change in sarcomere length [SL]) responses to electrical field stimulation were measured simultaneously in cardiomyocytes using an IonOptix system (Ionoptix LCC), as previously described (Schaible et al., 2016). Briefly, to measure the [Ca^2+^]_cyt_ response, isolated cardiomyocytes were loaded with 1 μM Fura‐2 AM for 10 min at 37°C. After this loading, the cardiomyocytes were washed two times, placed in a tissue chamber (0.5 ml), and perfused with Ca^2+^ Tyrode's solution kept at 37°C and aerated with a 95% O_2_–5% CO_2_ gas mixture. Evoked [Ca^2+^]_cyt_ responses in cardiomyocytes were measured using a dual‐excitation spectrofluorometer (IonOpitx). Fura‐2 fluorescence was excited using alternating 340 and 380 nm wavelengths, and emission fluorescence at 510 nm was detected using a photomultiplier tube. The [Ca^2+^]_cyt_ was determined based on calibration of the ratio of Fura‐2 fluorescence (*R* = 340/380 nm) using the equation described by Grynkiewicz et al. (1985) The dissociation constant (*K*_d_) was determined at 37°C based on an in vitro (cell‐free) calibration performed using Fura‐2 pentapotassium (Fura‐2 PP) salt in which *K*_d_ is reflected by the *x*‐intercept of the double‐log plot of free Ca^2+^ vs. Fura‐2 fluorescence. The isolated cardiomyocytes were stimulated using platinum wires positioned on the side of the chamber with 15 V 5 ms duration pulses delivered in 2 s duration trains at a frequency of 0.5 Hz using the MyoPacer stimulator (IonOptix). The cardiomyocytes were initially stimulated for 15 min to allow sufficient time for stabilization of [Ca^2+^]_cyt_ and contractile responses (baseline). The amplitude of evoked [Ca^2+^]_cyt_ responses was measured as well as the total the area under the curve of the transient response. In addition, the time to peak [Ca^2+^] and tau were determined. Contractile responses were assessed by measuring the extent and velocity of SL shortening in a region of interest within the cardiomyocyte encompassing at least seven sarcomeres and calculated based on the fast Fourier transform algorithm. Continuous measurements of SL were determined from rest (diastolic) to peak contractile (systolic) back to resting state during each stimulation cycle. In addition, the times to SL shortening peak 10%, 50%, and 90%, and times to SL relaxation 10%, 50%, and 90% were measured. Ten evoked [Ca^2+^]_cyt_ and SL responses were obtained at each time point in the experimental protocol (Figure 1). The sensitivity of the contractile response to [Ca^2+^]_cyt_ was determined from phase‐loop plots of [Ca^2+^]_cyt_ and SL responses. From these plots, the [Ca^2+^]_cyt_ at which 50% shortening and 50% relaxation occurred were determined as measures of myofilament Ca^2+^ sensitivity in cardiomyocytes.

### 2.4 Response to arginase inhibition

To determine the effects of arginase inhibition, [Ca^2+^]_cyt_ and SL responses of cardiomyocytes were measured before and after adding 10^−5^ M *S*‐[2‐boronoethyl]‐l‐cysteine (BEC; Calbiochem) to the perfusion solution. After determining the effect of BEC, the same cardiomyocytes were perfused with a NOS inhibitor, *N* ^G^‐nitro‐l‐arginine‐methyl ester (l‐NAME, 10^−4^ M) together with BEC (10^−5^ M). The concentrations of BEC and l‐NAME were determined based on previous reports demonstrating increasing and offsetting effects on contractility in isolated cardiomyocytes, respectively (Khan et al., 2012; Steppan et al., 2006). Cardiomyocytes were perfused with BEC and BEC + l‐NAME for 3 min, respectively, and [Ca^2+^]_cyt_ and SL responses of cardiomyocytes were measured during the last minute. Between exposures to BEC and BEC + l‐NAME, the cardiomyocytes were washed by perfusion with Tyrode solution for 7 min. The [Ca^2+^]_cyt_ and SL responses of separate untreated cardiomyocytes were used as time‐matched controls experiment was performed to determine the effect of time on [Ca^2+^]_cyt_ and SL responses (Figure 1).

### 2.5 Chemicals and solutions

BEC and l‐NAME were purchased from Calbiochem. Ca^2+^ Tyrode solution (in mM) consisted of 137 NaCl, 5.4 KCl, 0.5 MgCl_2_, 1.8 CaCl_2_, 0.33 NaH_2_PO_4_, 10.0 HEPES, and 10.0 glucose and was adjusted to pH 7.4 with NaOH. Enzyme solution consisted of zero‐Ca^2+^ Tyrode solution supplemented with 0.2 mM CaCl_2_, 0.6 mg/ml type II collagenase (Worthington), and 0.1 mg/ml protease with 1% bovine serum albumin. Kraft‐Brühe solution (in mM) contained 70 KOH, 50 l‐glutamic acid, 40 KCl, 0.5 MgCl_2_, 1 KH_2_PO_4_, 0.5 EGTA, 10 HEPES, 5 pyruvic acid, 5 Na_2_ATP, 5.5 glucose, 20 taurine, and 5 creatinine, and was adjusted to pH 7.38 with KOH. Cell medium (#6201; ScienCell) consisted of 500 ml of basal medium, 25 ml of fetal bovine serum (Cat. No. 0025), 5 ml of cardiac myocyte growth supplement (CMGS, Cat. No. 6252) and 5 ml of penicillin/streptomycin solution (P/S, Cat. No. 0503).

### 2.6 Statistical analysis

One or two cardiomyocytes were studied per animal and animals (*n* = 6 per group) were assigned to treatment and time‐matched control groups. Because there were differences in the number of cardiomyocytes studied per animal, inter‐animal variability was considered in the statistical/experimental design. In addition, at each time point, 10 evoked responses were obtained for each cardiomyocyte. Thus, variance across the responses in each cardiomyocyte was also considered in the statistical/experimental design. In a preliminary study, the mean and standard deviation (SD) of 10 evoked [Ca^2+^]_cyt_ and SL shortening responses were measured in untreated cardiomyocytes and used in a power analysis to determine the number of cardiomyocytes and animals required to measure an effect size of 20% (power = 80%, *α* = 0.05). The results of this study were based on an *n* = 6 animals per group, an *n* = 10 of cardiomyocytes per group and an *n* = 10 evoked response per cardiomyocyte. A mixed linear model analysis of variance was used to compare the effects of BEC−, BEC + l‐NAME (treatment group) and time (time‐matched controls) to baseline with animal as a random effect and treatment group, time and group‐by‐time as fixed effects. When variables showed significant differences between groups, post hoc analyses with Bonferroni correction were performed for the adjustment of multiple comparisons. Statistical analysis was performed using Prism 9.0.2 software (GraphPad). Results were presented as mean ± SD. A *p*‐value <0.05 was considered significant.

## 3 RESULTS

### 3.1 Evoked [Ca^2+^]_cyt_ responses

**FIGURE 2:**
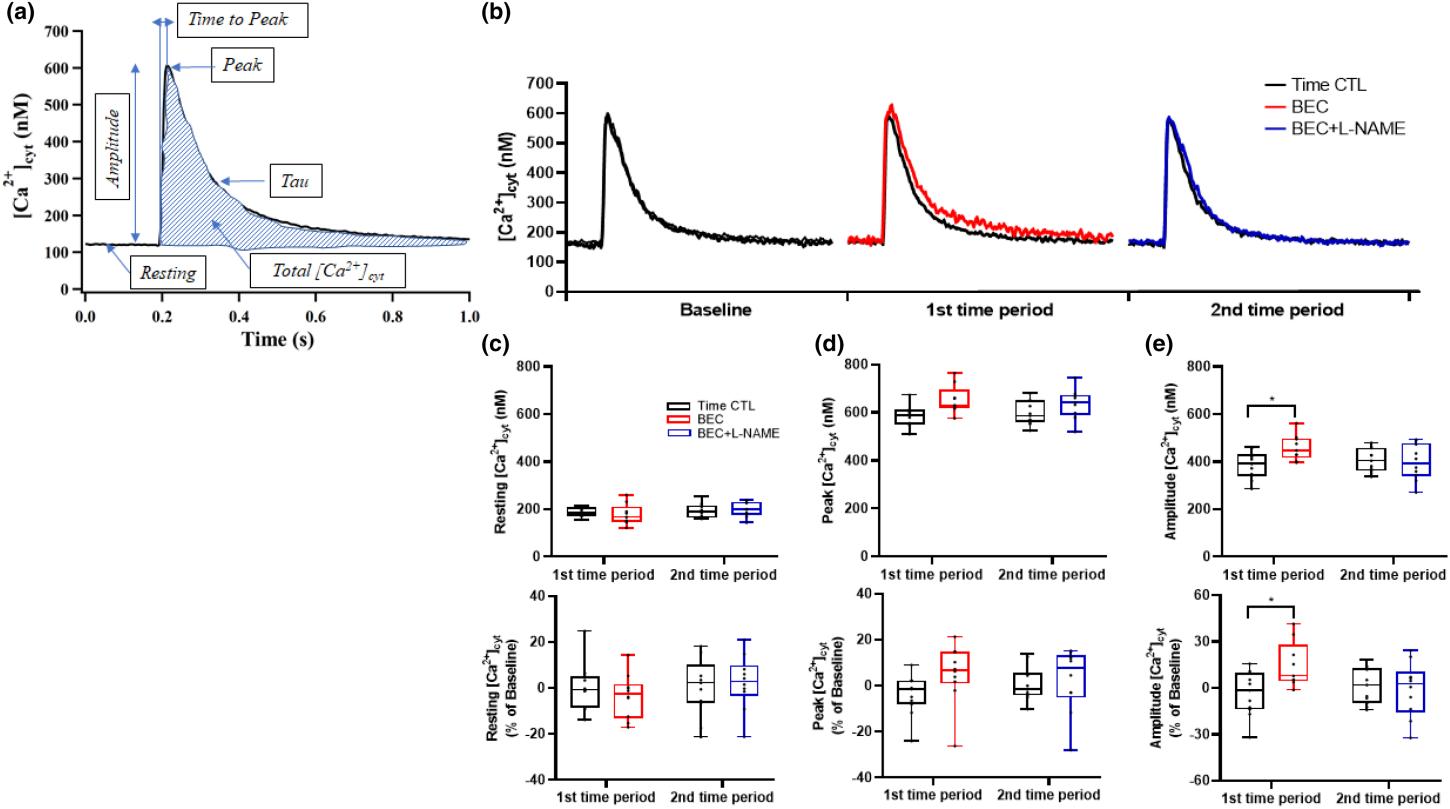
(a, b) Representative [Ca^2+^]_cyt_ response of cardiomyocytes to electrical field stimulation (0.5 Hz). (c) The resting and (d) peak [Ca^2+^]_cyt_ responses did not change across treatment groups. (e) The amplitude of evoked [Ca^2+^]_cyt_ responses increased following BEC treatment (*p < 0.05). This effect was mitigated following BEC + l‐NAME treatment (*p < 0.05). Time‐matched controls displayed no change in evoked [Ca^2+^]_cyt_ response. *n* = 6 treatment and *n* = 6 time‐matched controls. The evoked [Ca^2+^]_cyt_ response was averaged over 10–20 stimulation cycles. A mixed linear model analysis of variance (ANOVA) with Bonferroni correction was performed to compare the effects of BEC−, BEC + l‐NAME (treatment group) and time (time‐matched controls) to baseline. BEC, *S*‐[2‐boronoethyl]‐l‐cysteine; l‐NAME, *N* ^G^‐nitro‐l‐arginine‐methyl ester.

The resting [Ca^2+^]_cyt_ (no stimulation) remained constant at 180.6 ± 23.2 nM across the experimental period (14 min) and was comparable between experimental and time‐matched control groups (Figure 2a–c). The baseline peak [Ca^2+^]_cyt_ (maximum response) was 606.2 ± 28.5 nM and increased following BEC treatment, but the change across time did not reach statistical significance (*p* = 0.087) (Figure 2d). Compared to baseline, the amplitude of the evoked [Ca^2+^]_cyt_ response (peak [Ca^2+^]_cyt_ – resting [Ca^2+^]_cyt_) increased following BEC treatment (400.9 ± 21.4 nM vs. 441.2 ± 78.1 nM, *p* = 0.005), and this effect was mitigated following BEC + l‐NAME treatment. The amplitude of [Ca^2+^]_cyt_ did not change across time in the time‐matched controls (Figure 2e).

**FIGURE 3:**
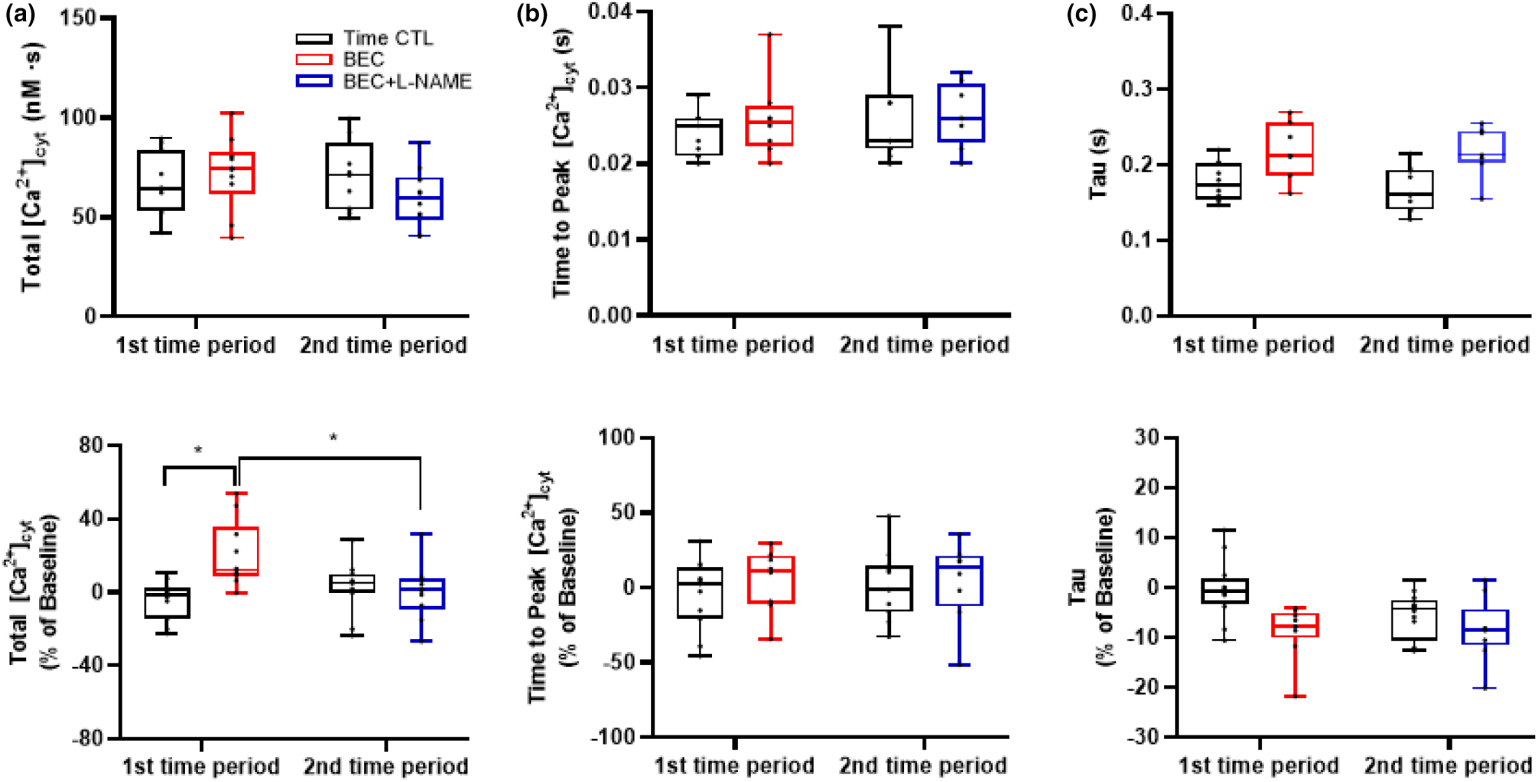
(a) The total amount of evoked [Ca^2+^]_cyt_ expressed in percent change from baseline increased following BEC treatment (*p < 0.05). This effect was mitigated following BEC + l‐NAME treatment (*p < 0.05). Time‐matched controls displayed no change in evoked [Ca^2+^]_cyt_ response. (b) Time to peak [Ca^2+^]_cyt_ and (c) tau were unaffected by treatment or time. *n* = 6 treatment and *n* = 6 time‐matched controls. The evoked [Ca^2+^]_cyt_ response was averaged over 10–20 stimulation cycles. A mixed linear model analysis of variance (ANOVA) with Bonferroni correction was performed to compare the effects of BEC−, BEC + L‐NAME (treatment group) and time (time‐matched controls) to baseline. BEC, *S*‐[2‐boronoethyl]‐l‐cysteine; l‐NAME, *N* ^G^‐nitro‐l‐arginine‐methyl ester.

The baseline total amount of evoked [Ca^2+^]_cyt_ was 60.7 ± 15.7 nM. Compared to baseline, the total amount of evoked [Ca^2+^]_cyt_ expressed in percent change from baseline increased following BEC treatment (Figure 3a; *p* = 0.002), and this effect was mitigated following BEC + l‐NAME treatment (*p* = 0.016). The total amount of evoked [Ca^2+^]_cyt_ remained constant across time in time‐matched control cardiomyocytes. Time to peak [Ca^2+^]_cyt_ and tau were not affected by treatment or time (Figure 3b,c; Table 1).

**TABLE 1:** Analysis of [Ca^2+^]_cyt_ and sarcomere length shortening

	Parameters	BEC	BEC + l‐NAME	*p* value
[Ca^2+^]_cyt_	Time to peak (s)	0.026 ± 0.005	0.026 ± 0.004	0.834
	(%baseline)	(95.0 ± 13.9)	(97.1 ± 10.4)	(0.750)
	Amplitude	441.2 ± 78.1	397.4 ± 75.9	0.220
	(%baseline)	(110.3 ± 20.3)	(99.1 ± 17.7)	(0.204)
	Tau	0.24 ± 0.06	0.24 ± 0.07	0.951
	(%baseline)	(91.2 ± 5.8)	(91.6 ± 6.8)	(0.895)
	Return velocity	5.611 ± 2.286	5.601 ± 2.540	0.994
	(%baseline)	(104.7 ± 21.3)	(104.1 ± 27.4)	(0.963)
SL shortening	Time to peak 10% (s)	0.011 ± 0.004	0.011 ± 0.005	0.834
	(%baseline)	(121.0 ± 63.0)	(123.8 ± 58.9)	(0.927)
	Time to peak 50% (s)	0.025 ± 0.009	0.025 ± 0.009	0.978
	(%baseline)	(106.7 ± 28.0)	(106.8 ± 27.0)	(0.989)
	Time to peak 90% (s)	0.054 ± 0.017	0.055 ± 0.025	0.910
	(%baseline)	(115.1 ± 23.6)	(115.3 ± 31.3)	(0.983)
	Time to baseline 10% (s)	0.105 ± 0.033	0.098 ± 0.029	0.657
	(%baseline)	(112.4 ± 12.4)	(105.2 ± 8.3)	(0.195)
	Time to baseline 50% (s)	0.132 ± 0.041	0.125 ± 0.037	0.712
	(%baseline)	(109.8 ± 17.3)	(103.6 ± 11.2)	(0.411)
	Time to baseline 90% (s)	0.275 ± 0.074	0.245 ± 0.111	0.684
	(%baseline)	(126.4 ± 49.0)	(91.1 ± 58.9)	(0.425)

*Note*: The results are presented as the mean ± standard deviation (*n* = 6).

Abbreviations: BEC, *S*‐[2‐boronoethyl]‐l‐cysteine; l‐NAME, *N* ^G^‐nitro‐l‐arginine‐methyl ester; SL, sarcomere length.

### 3.2 Extent of SL shortening

**FIGURE 4:**
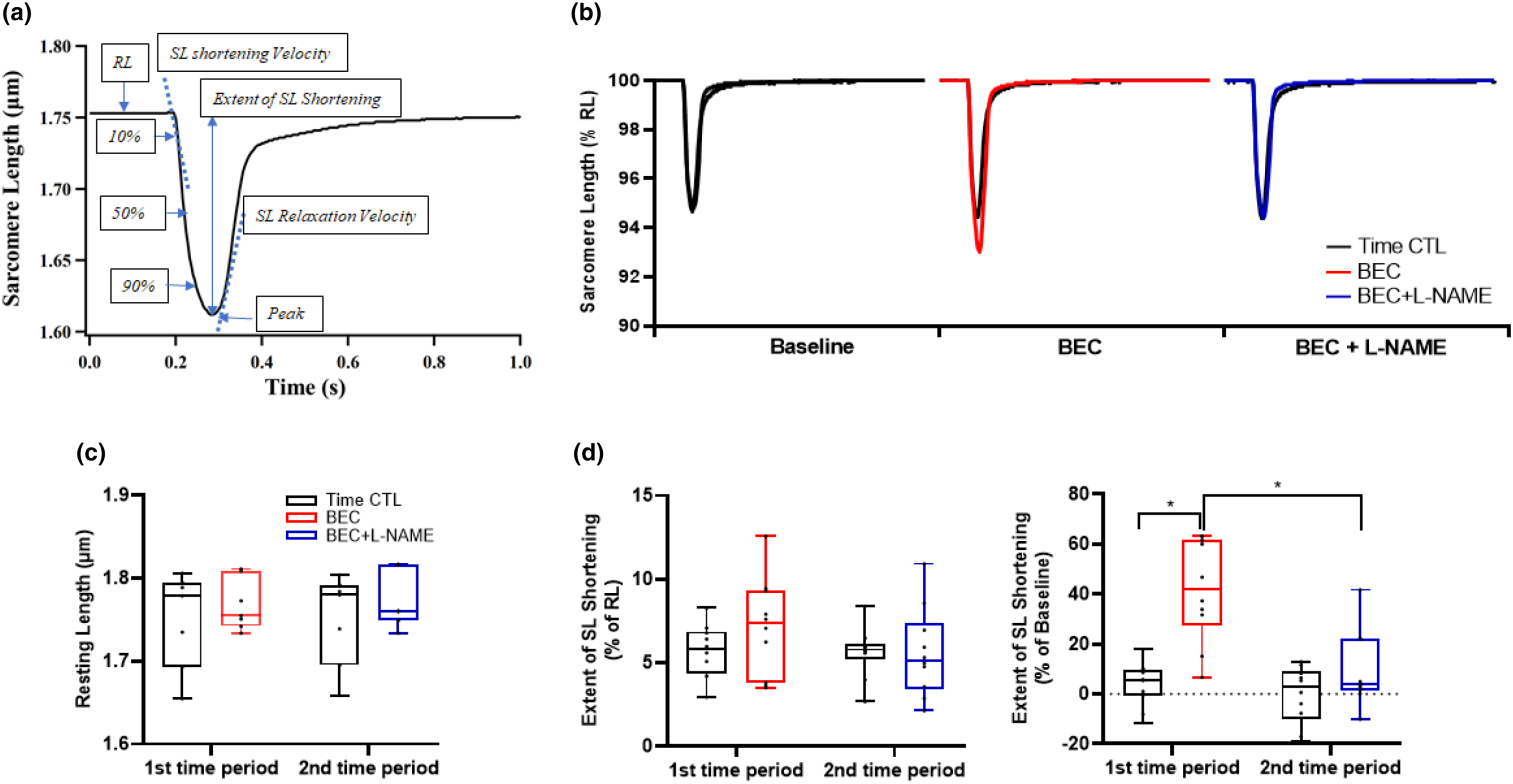
(a, b) Representative contractile response of cardiomyocytes to electric field stimulation (0.5 Hz). (c) The resting length (RL) was unaffected by treatment or time. (d) The extent of SL shortening expressed in percent change from baseline increased significantly following BEC treatment (*p < 0.05). This enhancement of contractile response was mitigated following BEC + l‐NAME treatment (*p < 0.05). Time‐matched controls displayed no change in contractile responses. *n* = 6 treatment and *n* = 6 time‐matched controls. The contractile response was averaged over 10–20 stimulation cycles. A mixed linear model analysis of variance (ANOVA) with Bonferroni correction was performed to compare the effects of BEC‐, BEC + l‐NAME (treatment group) and time (time‐matched controls) to baseline. BEC, *S*‐[2‐boronoethyl]‐l‐cysteine; l‐NAME, *N* ^G^‐nitro‐l‐arginine‐methyl ester; SL, sarcomere length.

The baseline resting SL was 1.8 ± 0.03 μm and was comparable across treatments and between treatment and time‐matched control groups (Figure 4a–c). The baseline extent of SL shortening induced by stimulation was 5.0 ± 1.9% of resting SL (Figure 4d). Compared to baseline, the extent of SL shortening expressed in percent change from baseline increased significantly following BEC treatment (Figure 4d; *p* < 0.001), whereas this enhancement of contractile response was mitigated following BEC + l‐NAME treatment (*p* = 0.001). Compared to baseline, the extent of SL shortening evoked by stimulation remained constant across time in the time‐matched control cardiomyocytes (Figure 4d).

### 3.3 Times to SL shortening and relaxation

**FIGURE 5:**
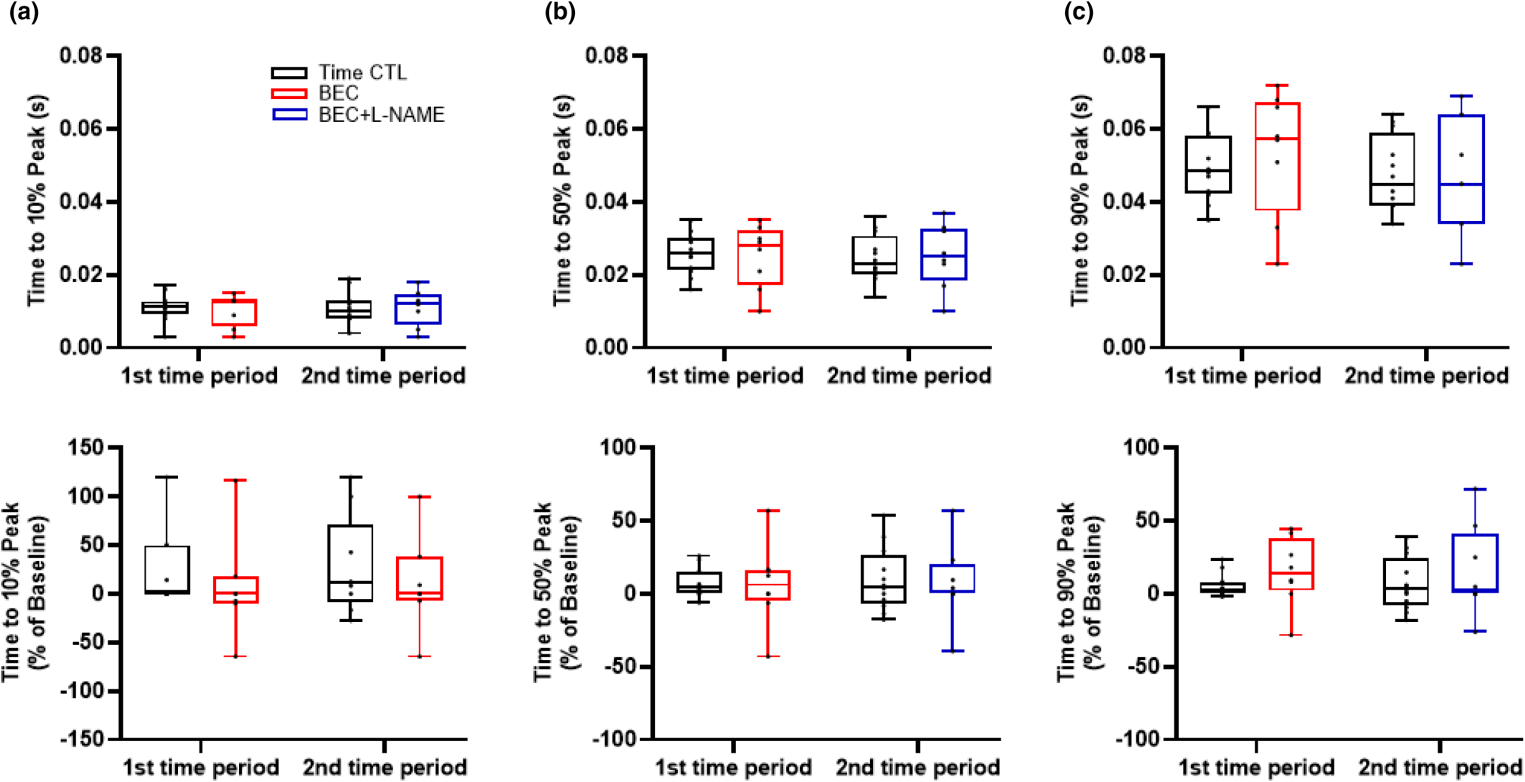
(a) Times to SL shortening peak 10%, (b) 50% and (c) 90% were not affected by treatment or time. *n* = 6 treatment and *n* = 6 time‐matched controls. The contractile response was averaged over 10–20 stimulation cycles. A mixed linear model analysis of variance (ANOVA) with Bonferroni correction was performed to compare the effects of BEC−, BEC + L‐NAME (treatment group) and time (time‐matched controls) to baseline. BEC, *S*‐[2‐boronoethyl]‐l‐cysteine; l‐NAME, *N* ^G^‐nitro‐l‐arginine‐methyl ester; SL, sarcomere length.

**FIGURE 6:**
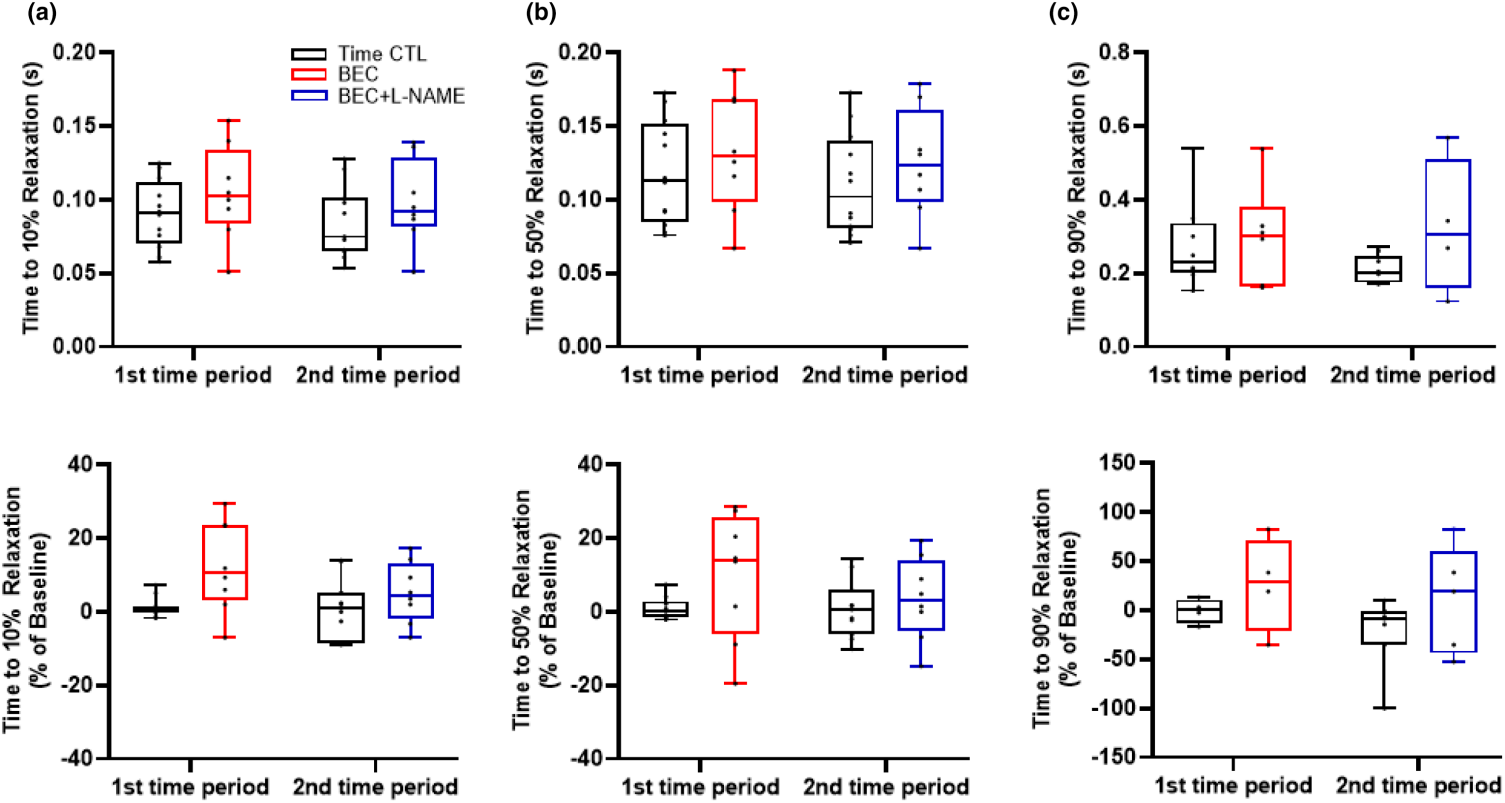
(a) Times to SL relaxation 10%, (b) 50%, and (c) 90% were not affected by treatment or time. *n* = 6 treatment and *n* = 6 time‐matched controls. The contractile response was averaged over 10–20 stimulation cycles. A mixed linear model analysis of variance (ANOVA) with Bonferroni correction was performed to compare the effects of BEC−, BEC + l‐NAME (treatment group) and time (time‐matched controls) to baseline. BEC, *S*‐[2‐boronoethyl]‐l‐cysteine; l‐NAME, *N* ^G^‐nitro‐l‐arginine‐methyl ester; SL, sarcomere length.

Times to SL shortening peak 10%, 50% and 90% (Figure 5a–c) and times to SL relaxation 10%, 50%, and 90% (Figure 6a–c) were not affected by treatment or time (Table 1).

### 3.4 Ca^2+^ sensitivity of contraction and relaxation

**FIGURE 7:**
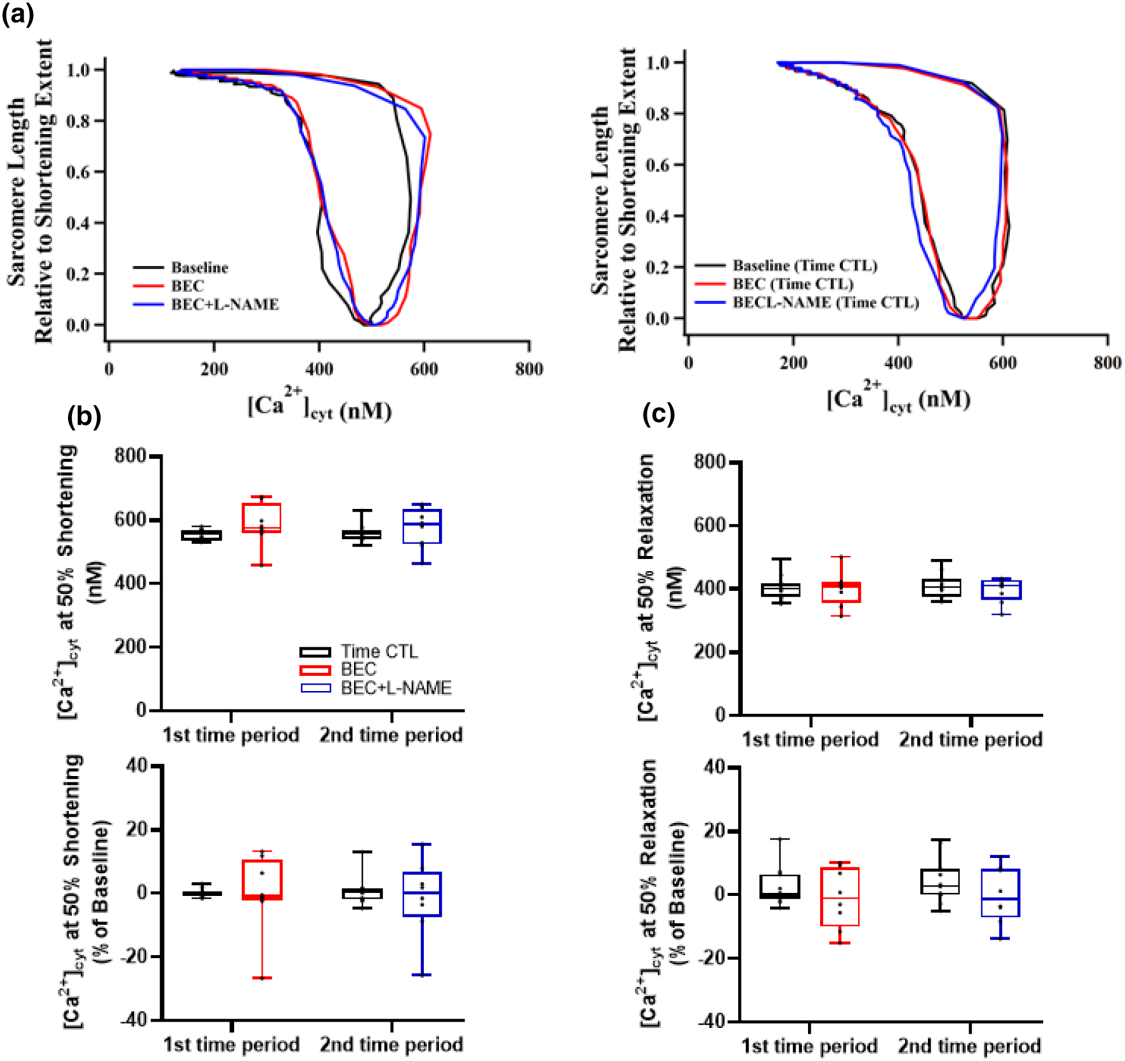
(a) Representative phase‐loop plot of [Ca^2+^]_cyt_ and sarcomere length response to electrical field stimulation. Left, treatment group; right, time matched controls. (b) [Ca^2+^]_cyt_ at which 50% shortening occurred was unaffected by treatment or time. (c) [Ca^2+^]_cyt_ at which 50% relaxation occurred was unaffected by treatment or time. *n* = 6 treatment and *n* = 6 time‐matched controls. The evoked [Ca^2+^]_cyt_ and contractile responses were averaged over 10–20 stimulation cycles. A mixed linear model analysis of variance (ANOVA) with Bonferroni correction was performed to compare the effects of BEC−, BEC + l‐NAME (treatment group) and time (time‐matched controls) to baseline. BEC, *S*‐[2‐boronoethyl]‐_L_‐cysteine; l‐NAME, *N* ^G^‐nitro‐l‐arginine‐methyl ester; SL, sarcomere length.

The Ca^2+^ sensitivity of the contractile response in cardiomyocytes was evaluated by comparing phase‐loop plots of evoked [Ca^2+^]_cyt_ and SL shortening/relaxation responses (Figure 7a). The baseline [Ca^2+^]_cyt_ at which 50% shortening occurred was 554.0 ± 44.4 nM (Figure 7b), while the baseline [Ca^2+^]_cyt_ at which 50% relaxation occurred were and 395.5 ± 42.6 nM (Figure 7c). Compared to baseline, the [Ca^2+^]_cyt_ at 50% contraction was not affected by either BEC or BEC + l‐NAME treatment (Figure 7b). Similarly, compared to baseline, the [Ca^2+^]_cyt_ at 50% relaxation was not affected by either BEC or BEC + l‐NAME treatment (Figure 7c). The [Ca^2+^]_cyt_ at 50% shortening and 50% relaxation did not across time in the time‐matched control group cardiomyocytes (Figure 7b,c).

## 4 DISCUSSION

The main finding of the present study was that an increase in NO following BEC‐induced arginase inhibition in isolated cardiomyocytes resulted in an increase in the total [Ca^2+^]_cyt_ response to electrical stimulation. As a result, both the extent and velocity of SL shortening increased after BEC treatment. These effects of BEC treatment were mitigated by l‐NAME mediated NOS inhibition. Importantly, BEC treatment did not affect Ca^2+^ sensitivity of the contractile response in cardiomyocytes.

### 4.1 Effect of NO on [Ca^2+^]_cyt_ and contractile responses

Enhanced NO production in cardiomyocytes by BEC treatment increased the amplitude and total amount of [Ca^2+^]_cyt_. The effects of NO on [Ca^2+^]_cyt_ and contractility depend on the specific isoform of NOS and its localization within the cardiomyocyte (Khan et al., 2003). Endothelial NOS (NOS3) is localized to sarcolemmal caveolae (Barouch et al., 2002), and NOS3‐generated NO activates cyclic guanosine monophosphate (cGMP)‐dependent pathways and negatively regulates L‐type Ca^2+^ channels, inhibiting cardiac contractility and β‐adrenergic inotropy (Hare, 2003; Steppan et al., 2006). In contrast, neuronal NOS (NOS1) is localized to the SR (Barouch et al., 2002), and modulates the release and sequestration of Ca^2+^ by the SR by targeting proteins such as RyR and phospholamban (Ziolo et al., 2008). NOS1‐generated NO modulates nitrosylation of RyR, increasing the probability of RyR opening and Ca^2+^ release from the SR (Khan et al., 2003). Sequestration of the [Ca^2+^]_cyt_ response is due in part to SERCA mediated transport of Ca^2+^ from the cytosol into the SR. The activity of SERCA is modulated by phosphorylation of phospholamban, which is increased by NO. Thus, NO increases SERCA activity, which would increase the rate and amount of [Ca^2+^]_cyt_ sequestration by the SR, enhancing cardiac contractility (Movsesian & Schwinger, 1998; Wang et al., 2008). In the present study, the effect of BEC on [Ca^2+^]_cyt_ and cardiac contractility in isolated cardiomyocytes was minor, but statistically significant.

### 4.2 Effect of arginase inhibition on [Ca^2+^]_cyt_ response

One regulatory mechanism for NOS is substrate bioavailability. Arginase is a critical regulator of NO synthesis as it reciprocally regulates NOS activity by competing for a common substrate, l‐arginine (Durante et al., 2007). Arginase II is the predominant isoform of arginase in rodent hearts and coimmunoprecipitates only with NOS1 and not with NOS3. The adjacent intracellular localization of arginase II and NOS1 suggests a close molecular interaction (Steppan et al., 2006). Although some studies have reported that arginase increases cardiac contractility in a NO‐dependent manner (Khan et al., 2012; Steppan et al., 2006), in those studies, the effect of arginase on evoked [Ca^2+^]_cyt_ responses has not been investigated concurrently. Considering that NO increases both the release and sequestration of Ca^2+^ by the SR (Khan et al., 2003), we hypothesized that arginase inhibition would increase cardiac contracility either by increasing [Ca^2+^]_cyt_ or enhancing Ca^2+^ sensitivity of the contractile response.

Arginase inhibition induces NOS1 activation and increases NO production (Steppan et al., 2006), which may increase the [Ca^2+^]_cyt_ response to electrical stimulation by increasing SR Ca^2+^ release via RyR or SR Ca^2+^ stores by increasing SERCA activity (Ziolo et al., 2008). In the present study, BEC significantly increased both amplitude and the total amount of evoked [Ca^2+^]_cyt_ to electrical stimulation. Importantly, l‐NAME mitigated the BEC‐induced increase in evoked [Ca^2+^]_cyt_ responses, indicating that arginase affected [Ca^2+^]_cyt_ through a NOS‐dependent mechanism. Our findings are consistent with a previous study reporting that both in vivo and isolated myocytes from NOS1 knockout (NOS1^−/−^) mice exhibited reduced SR Ca^2+^ release and SR Ca^2+^ stores in response to increasing frequency of stimulation (Khan et al., 2003). In contrast, a study using feline isolated cardiomyocytes reported that arginase inhibition with BEC reduced the peak [Ca^2+^] and SL shortening (Jung et al., 2006). The predominant isoform of arginase is arginase II in rodent hearts, which coimmunoprecipitates with NOS1 and positively modulates myocardial contractility, whereas only arginase I was exclusively expressed in feline isolated cardiomyocytes (Jung et al., 2006; Pernow & Jung, 2013). The negative effects of arginase inhibition on [Ca^2+^]_cyt_ and contractile responses in feline cardiomyocytes were associated with increased cGMP production, and guanylate cyclase inhibitor reversed these effects (Jung et al., 2006). Activation of cGMP‐dependent pathways negatively modulates β‐adrenergic responsiveness and cardiac contractility (Layland et al., 2002).

### 4.3 Effect of arginase inhibition on contractile responses

Arginase influences cardiac contractility by reciprocally regulating NOS and NO (Khan et al., 2012; Steppan et al., 2006). Arginase inhibition increases NO bioavailability, restores NOS coupling and NO production, and thus increases basal contractility in cardiomyocytes (Steppan et al., 2006). We also found that arginase inhibition enhanced cardiac contractility by increasing the extent of SL shortening to electrical stimulation. In addition, a NOS inhibitor mitigated BEC‐induced contractile responses, reflecting the reciprocal regulation of NOS by arginase. In NOS1^−/−^ mice, the responses to both increasing frequency of stimulation and to β‐adrenergic stimulation are depressed (Barouch et al., 2002; Wang et al., 2008). The positive inotropic effect of NO on cardiac contractility is constitutively dependent on [Ca^2+^]_cyt_ (Heinzel et al., 2008; Yang et al., 2004). Our study also showed that enhanced contractility by arginase inhibition was associated with increased [Ca^2+^]_cyt_ response. In an animal study, an increase in arginase was associated with aging‐related reduced cardiac contractility (Khan et al., 2012). Cardiomyocytes from older animals exhibited reduced contractile responses compared to cardiomyocytes from younger animals, which was associated with increased activity and expression of arginase and disrupted pattern between arginase II and NOS in aged cardiomyocytes. Arginase inhibition restored NO production and contractile responses in cardiomyocytes from older animals.

### 4.4 Effect of arginase inhibition on Ca^2+^ sensitivity

In addition to modulating Ca^2+^, NO influences cardiac contractility through its role in maintaining levels of superoxide anions in cells (Khan et al., 2004). NOS1 and xanthine oxidoreductase (XOR) are expressed within proximity to the SR. XOR produces a superoxide anion, which diffuses out of the SR and reduces Ca^2+^ sensitivity of cardiomyocytes (Berry & Hare, 2004). NO directly reacts with superoxide anions to inhibit its release from the SR, thereby reducing its inhibition of cardiac contractility (Beckman & Koppenol, 1996). Considering that NO negatively regulates inhibition of cardiac excitation‐contraction coupling by XOR, we hypothesized that arginase inhibition might affect cardiac contractility by altering the Ca^2+^ sensitivity of cardiomyocytes. In the present study, arginase inhibition by BEC treatment did not affect Ca^2+^ sensitivity as reflected by the [Ca^2+^] at which 50% contraction and 50% relaxation occurred. The lack of an effect of arginase inhibition on Ca^2+^ sensitivity in the present study might reflect the absence of oxidative stress. Peroxynitrite, an indicator of oxidative stress (Beckman & Koppenol, 1996), increased arginase activity and contributed cardiac dysfunction in endotoxemia rats (Khadour et al., 2002). In this regard, the effect of arginase inhibition on cardiac contractility in conditions with increased oxidative stress would merit further investigation.

In addition to the previously known effect of arginase inhibition on cardiac contractility, we demonstrate that the enhanced contractility induced by arginase inhibition was associated with an increase in total evoked [Ca^2+^]_cyt_ to electrical stimulation. Although we did not measure NO production, the relationship between arginase inhibition and NO production has been well documented (Khan et al., 2012; Steppan et al., 2006). Impaired SR Ca^2+^ cycling, including reduced Ca^2+^ uptake, decreased SR Ca^2+^ sequestration, and defective Ca^2+^ release from the SR, are hallmarks of heart failure and contribute to its pathophysiology and progression (Kho et al., 2012). Arginase upregulation and the resulting decreased bioavailability of NOS have been reported to contribute to the pathophysiology of disease processes in which NO signaling is dysregulated, such as aging (Berkowitz et al., 2003), hypertension (Demougeot et al., 2005), and endotoxemia (Khadour et al., 2002). Arginase inhibition has shown beneficial effects on SR Ca^2+^ cycling and cardiac contractility and may be a novel target for therapy in NO related‐cardiac dysfunction.

## 5 CONCLUSION

Arginase inhibition by BEC significantly increased the amplitude and the total amount of evoked [Ca^2+^]_cyt_ to electrical stimulation. It enhanced myocardial contractility by increasing the extent and velocity of SL shortening in cardiomyocytes, but did not affect Ca^2+^ sensitivity. These effects were mitigated by NOS inhibition, supporting a role of NO on [Ca^2+^]_cyt_ and contractile responses. Our findings suggest the potential of arginase inhibition in the treatment of NO‐related cardiac dysfunction.

## FUNDING INFORMATION

There is no funding to be declared for this study.

## CONFLICT OF INTEREST

The authors declare no competing interests.

## ETHICS STATEMENT

All animal procedures were approved by the Mayo Clinic Institutional Animal Care and Use Committee (IACUC).
